# Pedagogical Concerns in Sports and Physical Education for Child Growth and Health Promotion

**DOI:** 10.3390/ijerph19138128

**Published:** 2022-07-02

**Authors:** Luís Branquinho, Pedro Forte, Ricardo Ferraz

**Affiliations:** 1 Department of Sports, Higher Institute of Educational Sciences of the Douro, 4560-708 Penafiel, Portugal; pedromiguel.forte@iscedouro.pt; 2 Research Centre in Sports Sciences, Health Sciences and Human Development (CIDESD), 6201-001 Covilhã, Portugal; ricardompferraz@gmail.com; 3 CI-ISCE, ISCE Douro, 4560-708 Penafiel, Portugal; 4 Department of Sport Sciences and Physical Education, Polytechnic Institute of Bragança (IPB), 5300-253 Bragança, Portugal; 5 Department of Sports Sciences, University of Beira Interior, 6201-001 Covilhã, Portugal

## 1. Introduction

Participation in physical activities plays an important role in and positively influences health and well-being. However, levels of physical activity continue to present worrying levels, with an estimated 2 million people currently dying worldwide as a result of sedentary and inactive lifestyles. For these reasons, it is essential to educate the new generations with healthy habits and lifestyles that can prevail from childhood to adulthood. In fact, childhood is a moment of transformation for children. The discovery of the body and its infinite possibilities of movement, combined with the relationships established with people and the environment, enhance the connection between children and physical activity. It is expected that through this type of stimuli, during growth, a solid cognitive and motor repertoire will be developed that will enable them to overcome challenges imposed by everyday life. Even so, due to an increasingly industrialized society, children have recurrently poor rates of motor literacy and high levels of sedentary lifestyles, which translate into an abrupt increase in childhood obesity. In addition, it is currently known that the development of motor skills is directly related to the general development of children, and for these reasons, its use is essential for sustained growth and development. Physical education classes are the ideal context for promoting healthy habits and lifestyles and therefore cannot be neglected. Nowadays, children enter schools at an early age, which is why it is essential that the teachers responsible for guiding the practice of physical activity provide them with adjusted and creative pedagogical programs and with adequate motor stimulation that enables motor and cognitive development.

## 2. Perspectives on Physical Activity and Health during Childhood

The concepts of physical activity and quality of life are inseparable and have been the subject of analysis for centuries. In fact, since the 19th century, the importance of physical activity for the growth and sustained development of children has been reported [1]. Although several indicators point to physical activity as one of the main enhancers for the development of children, modern society seems to continue to neglect its importance [2], and this is one of the biggest challenges for children in the 21st century. Children should be able to be physically active in order to enhance their motor development and discover the taste for physical activity that can emerge from the understanding of the relationship between movement and well-being [3]. Health benefits such as increased aerobic fitness [4], reduced cardiovascular risk factors [5], increased muscle strength [6], and improved mental health [7] and quality of life in general [8,9] have also been associated with physical activity. For these reasons, previous studies recommend 60 min a day of moderate and vigorous physical activity for young people aged between 5 and 17 years of age [4,10]. Sedentary habits in childhood are a systemic problem that has gained strength through globalization and economic growth [11,12]. Nowadays, compliance with the daily recommendations for physical activity only occurs through concerted efforts by entities that actively participate in children’s daily lives (i.e., schools, leisure time, among others) and that provide a set of spaces, equipment, and human resources that facilitate the practice of physical activity in these populations [13]. Thus, the idea of the importance of educators and teachers from childhood seems to be cemented so that they can comply with the daily recommendations of physical activity, and more investigations on these topics are necessary. Instilling positive and proactive attitudes towards physical activity seems to be as important as providing adequate moments for daily and regular practice [14]. In this sense, the best strategies to combat the scourge of children’s sedentary lifestyle need to be clarified in the literature. Although the introduction of physical activity and healthy lifestyle habits into a child’s daily life can be carried out in several ways, it is widely accepted that this should be a priority in early childhood education [15,16]. There is an urgent need to place children as the main focus of analysis so that there is a distinct understanding of the different perspectives and experiences to promote physical activity and healthy lifestyle habits [17], and therefore, more investigations are needed in this regard.

## 3. The Importance of Physical Education in Child Growth

Physical activity is a form of education that uses movement as a pedagogical material, with a view to organizing and improving children’s motor, psychological, and social behavior [18]. The structured development of children through physical activity is an emerging topic in contemporary society and therefore continues to need extensive investigation. Until now, it is known that a child’s development is directly dependent on the motricity that judiciously modifies the nervous system [19]. Children attend the first years of school, during which the sensitive stages of development occur [20], and the inclusion of physical education in the curriculum allows for harmonious multilateral and interdisciplinary development, enabling the development of motor skills and basic skills [21].

The discipline of Physical Education is an integral and mandatory part of the school curriculum, and one of its main purposes is the promotion of active and healthy lifestyles [22,23]. Physical education can positively contribute to the physical (e.g., motor skills and physical fitness), social (e.g., cooperation, solidarity), affective (e.g., self-esteem, attitudes), and cognitive (e.g., knowledge, concentration) development of children and youth [24]. These domains, in addition to being fundamental in the healthy development of children and adolescents, can be assumed as predictors of regular participation in physical activity [24,25]. For these reasons, it is essential that teachers and researchers adopt and create creative and playful methodologies that captivate children by encouraging a taste for physical activity from an early age. Some studies and books have explored these topics [26,27,28], but more research is still needed on new programs and methodologies that can be used in the context of physical education for children’s structured (i.e., physical and cognitive) development.

The school context has the potential to influence children to adopt an active lifestyle, through inclusive and recognized quality physical education programs that promote the development of the motor skills necessary for an excited and rewarding participation in physical and sports activities throughout life [23,29,30].

In this sense, it is important to understand the context of the transition from childhood to adolescence and how barriers and facilitators of physical activity can be shaped by circumstances and determinants for the existence of different levels of motor and cognitive development among children and young people. Previous studies reported that some of the main factors that contributed to young people being physically inactive were: personal factors (i.e., motivation, concerns about appearance) and negative experiences in physical education (i.e., lack of motivation for the programs addressed). On the other hand, other evidence reports that access to physical activity programs, fun, increased perceived competence, and support from friends and family were seen as facilitators.

Based on changes in lifestyles that may have occurred in recent years (i.e., COVID-19 pandemic) and the fact that there is no generalized information for all countries, it is necessary to update and systematize knowledge through recent qualitative studies that emphasize children’s and adolescents perspectives on physical activity [31,32].

In fact, due to its contribution to quality of life, growth, and for health in general [4,8,13], physical activity is a component fundamental of a healthy lifestyle, constituting its promotion, a public health priority since childhood, and therefore, more research is still needed that reports emerging strategies on sport pedagogy and physical education practices for health.

## References

[1] Pangrazi R.P., Beighle A. (2020). Dynamic Physical Education for Elementary School Children.

[2] Le Masurier G., Corbin C.B. (2006). Top 10 Reasons for Quality Physical Education. J. Phys. Educ. Recreat. Danc..

[3] Maughan B., Little M. (2017). Child Development.

[4] Janssen I., LeBlanc A.G. (2010). Systematic Review of the Health Benefits of Physical Activity and Fitness in School-Aged Children and Youth. Int. J. Behav. Nutr. Phys. Act..

[5] Andersen L.B., Riddoch C., Kriemler S., Hills A. (2011). Physical Activity and Cardiovascular Risk Factors in Children. Br. J. Sports Med..

[6] Fritz J., Rosengren B.E., Dencker M., Karlsson C., Karlsson M.K. (2016). A Seven-year Physical Activity Intervention for Children Increased Gains in Bone Mass and Muscle Strength. Acta Paediatr..

[7] Lubans D., Richards J., Hillman C., Faulkner G., Beauchamp M., Nilsson M., Kelly P., Smith J., Raine L., Biddle S. (2016). Physical Activity for Cognitive and Mental Health in Youth: A Systematic Review of Mechanisms. Pediatrics.

[8] Gopinath B., Hardy L.L., Baur L.A., Burlutsky G., Mitchell P. (2012). Physical Activity and Sedentary Behaviors and Health-Related Quality of Life in Adolescents. Pediatrics.

[9] Gu X., Chang M., Solmon M.A. (2016). Physical Activity, Physical Fitness, and Health-Related Quality of Life in School-Aged Children. J. Teach. Phys. Educ..

[10] World Health Organization (2010). Global Recommendations on Physical Activity for Health.

[11] Malik V.S., Willett W.C., Hu F.B. (2013). Global Obesity: Trends, Risk Factors and Policy Implications. Nat. Rev. Endocrinol..

[12] Caballero B. (2007). The Global Epidemic of Obesity: An Overview. Epidemiol. Rev..

[13] Kasser S.L., Lytle R.K. (2013). Inclusive Physical Activity.

[14] Donnelly F.C., Mueller S.S., Gallahue D.L. (2016). Developmental Physical Education for All Children: Theory into Practice.

[15] Wright P.M., Stork S. (2013). Recommended Practices for Promoting Physical Activity in Early Childhood Education Settings. J. Phys. Educ. Recreat. Danc..

[16] Vidoni C., Ignico A. (2011). Promoting Physical Activity during Early Childhood. Early Child Dev. Care.

[17] O’Sullivan M., MacPhail A., Bailey R. (2010). Young People’s Voices in Physical Education and Youth Sport.

[18] Neto C. (2015). Child Motorcycle and Initial Training of Physical Education Teachers. E-BALONMANO COM.

[19] Anghelache V. (2020). Intellectual Development and Learning at Early Age. A Theoretical Perspective. Proceedings of the 4th International Scientific Conference SEC-IASR 2019.

[20] Rocha L., Campos C., Rocha C. (2003). A Educação Física No Jardim de Infância e No 1° CEB: Características e Contextos de Formação. Educ. Apprendere.

[21] Rus C.M., Talaghir L.-G., Iconomescu T.-M., Petrea R.G. (2019). Curriculum Changes in Secondary School Physical Education and Sport Subject in the Romanian Education System. Rev. Cercet. Interv. Soc..

[22] Corbin C.B. (2002). Physical Education as an Agent of Change. Quest.

[23] Tappe M.K., Burgeson C.R. (2004). Physical Education: A Cornerstone for Physically Active Lifestyles. J. Teach. Phys. Educ..

[24] Bailey R., Armour K., Kirk D., Jess M., Pickup I., Sandford R. (2009). The Educational Benefits Claimed for Physical Education and School Sport: An Academic Review. Res. Pap. Educ..

[25] Batista M.B., Romanzini C.L.P., Barbosa C.C.L., Blasquez Shigaki G., Romanzini M., Ronque E.R.V. (2019). Participation in Sports in Childhood and Adolescence and Physical Activity in Adulthood: A Systematic Review. J. Sports Sci..

[26] Syrmpas I., Digelidis N., Watt A., Vicars M. (2017). Physical Education Teachers’ Experiences and Beliefs of Production and Reproduction Teaching Approaches. Teach. Teach. Educ..

[27] Aktop A., Karahan N. (2012). Physical Education Teacher’s Views of Effective Teaching Methods in Physical Education. Procedia-Social Behav. Sci..

[28] Metzler M. (2017). Instructional Models in Physical Education.

[29] Pate R.R., Davis M.G., Robinson T.N., Stone E.J., McKenzie T.L., Young J.C. (2006). Promoting Physical Activity in Children and Youth: A Leadership Role for Schools: A Scientific Statement from the American Heart Association Council on Nutrition, Physical Activity, and Metabolism (Physical Activity Committee) in Collaboration with the Councils on Cardiovascular Disease in the Young and Cardiovascular Nursing. Circulation.

[30] Sallis J.F., McKenzie T.L. (1991). Physical Education’s Role in Public Health. Res. Q. Exerc. Sport.

[31] Foster C., Cowburn G., Allender S. (2007). The Views of Children on the Barriers and Facilitators to Participation in Physical Activity: A Review of Qualitative Studies. Lond. Natl. Inst. Health Clin. Excell. (NICE Public Health Collab. Centre-Physical Act.).

[32] Allender S., Cowburn G., Foster C. (2006). Understanding Participation in Sport and Physical Activity among Children and Adults: A Review of Qualitative Studies. Health Educ. Res..

